# Competing Interactions in DNA Assembly on Graphene

**DOI:** 10.1371/journal.pone.0018442

**Published:** 2011-04-12

**Authors:** Saliha Akca, Ashkan Foroughi, Daniel Frochtzwajg, Henk W. Ch. Postma

**Affiliations:** Department of Physics and Astronomy, California State University Northridge, Northridge, California, United States of America; University of Helsinki, Finland

## Abstract

We study the patterns that short strands of single-stranded DNA form on the top graphene surface of graphite. We find that the DNA assembles into two distinct patterns, small spherical particles and elongated networks. Known interaction models based on DNA-graphene binding, hydrophobic interactions, or models based on the purine/pyrimidine nature of the bases do not explain our observed crossover in pattern formation. We argue that the observed assembly behavior is caused by a crossover in the competition between base-base pi stacking and base-graphene pi stacking and we infer a critical crossover energy of 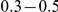 eV. The experiments therefore provide a projective measurement of the base-base interaction strength.

## Introduction

A thorough understanding of DNA binding to graphene is at the heart of interpreting DNA interactions with graphene-like substances. This is relevant to efforts of using DNA to sort carbon nanotubes, which may be thought of as folded up sheets of graphene [1]–[3], DNA sequencing using graphene [4], and DNA sensing with carbon nanotubes [5], [6], chemically-converted graphene [7], and graphene [8]–[10]. Here, we elucidate a crossover mechanism in interaction strengths between base-base binding and base-graphene binding.

## Results

**Figure 1:**
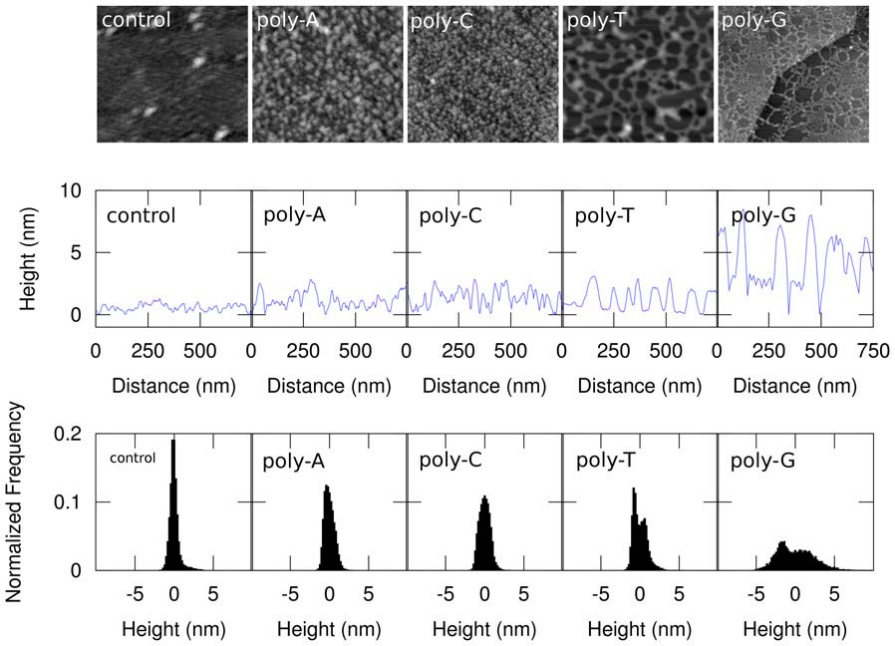
Atomic Force Micrographs of graphene with and without ssDNA, line scans, and histograms. (Top row) Atomic Force Micrographs of the height of the control experiment, poly-A, C, T, and G, respectively. All images are 750×750 nm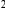, except for poly-G, which is 5×5 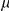
m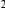. (Middle row) Representative line scan of height of the images above. (Bottom row) Histograms of the height as recorded in the AFM images corresponding to the AFM images in the top row as indicated. All histograms and line scans are over the entire images surface area, except poly-G, which is analyzed in the lower right corner to exclude the effect of the graphite step edge. The histograms and line scans share the vertical scale with the left-most graph.

Images of the assembled DNA on graphite are presented in figure 1. The line scans indicate that the control experiment shows a rather flat surface with a root mean square (RMS) height of 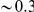 nm with a handful of scattered particles, while the poly-A and C are more rough with an RMS height of 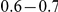 nm. In contrast, the line scans of the poly-T and G show a flat surface with a similar roughness as the control, interrupted by high and wide features. The narrow peak in the histogram corresponding to the image of the control is indicative of the natural corrugation of graphene, combined with a small layer of contamination from the buffer and deionized water, and environmetal and instrument fluctuations. The wider tail of the histogram on the right side of the control's histogram peak is caused by the small number of larger contamination particles that are visible in the image. In contrast, the wider peaks in the histograms for poly-A and C have no such wider tail and are symmetric. These histograms are indicative of the clustering of DNA into small tightly-packed spherical particles. Finally, the histograms for poly-T and G show two peaks, the lower of which corresponds to the graphene surface and the higher corresponds to the DNA.

Comparison of the control experiment with the ssDNA-deposited samples indicates that the extra features visible in the latter images are ssDNA that has assembled on the graphene surface. The poly-A and C DNA has formed a tightly-packed coverage of apparent spherical particles. In contrast, the poly-T and G has formed a stretched-out network on the surface with gaps in between the network strands. From the histogram, we find that the poly-T network strands are 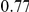 nm higher than the exposed flat areas in between. Because this is comparable to the single-nucleotide width of 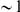 nm, we conclude that the network is made of single strands of DNA and the flat areas in between are exposed graphene surfaces. This is supported by the networked poly-G sample where the flat areas that are crossing the graphite step edge have a step edge that appears to be a continuation of the step edge that spans the image from the lower left to upper right corner. The apparent height is slightly smaller then expected and we anticipate this is due to the DNA being compressed by the tapping AFM tip as occurs with carbon nanotubes as well [11]. The poly-G network is 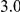 nm higher than the graphite surface. From this, we concluded that the poly-G network is most likely made of several strands packed together in a parallel manner, or this is possibly an indicator of Guanine tetraplex formation [12]. Note that we do not observe DNA assembling preferentially onto graphite step edges, emphasizing that DNA predominantly interacts with the top graphene surface.

## Discussion

It is clear that the DNA assembles in two distinct manners on the graphene surface. The poly-A and C have formed spherical particles, while the poly-T and G have formed a network on the surface. Several mechanisms have been suggested to play a role in DNA-graphene interaction, e.g. hydrogen bonding, base stacking, electrostatic, van der Waals, and hydrophobic interactions [13]. Note that in the experiments reported here, we have not applied any potential to facilitate adsorption, so the main mechanism is free adsorption [13]–[16]. Here, we first discuss candidate mechanisms for these two distinct assembly types and since they do not explain our observations, we propose a new model based on competing pi stacking interactions. The candidate mechanisms are summarized in table 1.

**Table 1:** Comparison of candidate interaction mechanisms and summary of the data.

Base	Network/Sphere	Type	DNA graphene interaction [17]–[19]	Hydrophobic Interaction [21]	Number of Hydrogen Bonds	Bond length (nm) [25]
A	sphere	purine	+	+	2	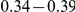
C	sphere	pyrimidine	-	–	3	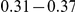
G	network	purine	++	++	3	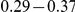
T	network	pyrimidine	+	-	2	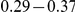

The ranking of interaction strengths and bond lenghts are derived from the noted references and are further discussed in the text.

### DNA - graphene binding

The main interaction mechanism of DNA interaction with graphene is through pi stacking [17], which is also the root cause of ssDNA binding more strongly then dsDNA to graphene [13]. It was found that G binds most strongly to graphene, whereas A, T, and C have similar binding strength (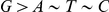) [17]. In two other studies, it was found that G 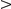 A 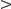 T 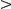 C [18], [19]. If the DNA-graphene interaction were the dominant factor in determining whether DNA assembles in networks or spheres, one would expect that A and T would then assemble in a similar manner. This clearly contradicts our observations, so we conclude that the DNA-graphene binding strength by itself is not responsible for the distinct assembly behavior we observe.

### Effect of hydrophobicity of DNA nucleobase

It has been argued that the hydrophobicity of the nucleobases plays a role in DNA-graphene interaction [13], [14], [20]. Guanine is the most hydrophobic, and in decreasing order of hydrophobicity, the bases are Adenine, Thymine, and Cytosine [21] (G 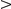 A 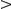 T 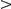 C). From figure 1, however, our experiments indicate that A is similar to C, and T is similar to G (A 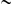 C, T 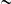 G). If the bases' hydrophobicity were responsible for the observed behavior, then A should be more similar to G then T is. In addition, C should be more similiar to T than A is. This is clearly in contradiction with our observations, so we conclude that the DNA's hydrophobicity does not play a disinguishable role in the observed DNA on graphene assembly.

### Number of hydrogen bonds

Note that the assembly behavior does not follow a pattern that correlates with the number of hydrogen bonds of the unhybridized bases. A and T have 2 hydrogen bonds, whereas C and G have 3. If the number of hydrogen bonds were the root cause of the different assembly behavior, then one would expect A and T to assemble similarly to each other. This is clearly in contradiction with our observations.

### Purine vs. Pyrimidine

It is especially interesting to note that the similarity in assembly behavior does not follow the purine-pyrimidine hierarchy of the bases, in contrast to what may be expected [22]. A and G would then assemble in a similar manner because they are purines and C and T would assemble similarly because they are pyrimidines. However, since our observations do not follow the pyrine/pyrimidine hierarchy, we conclude that this is not the mechanism that determines whether DNA assembles into spheres or a network.

**Figure 2:**
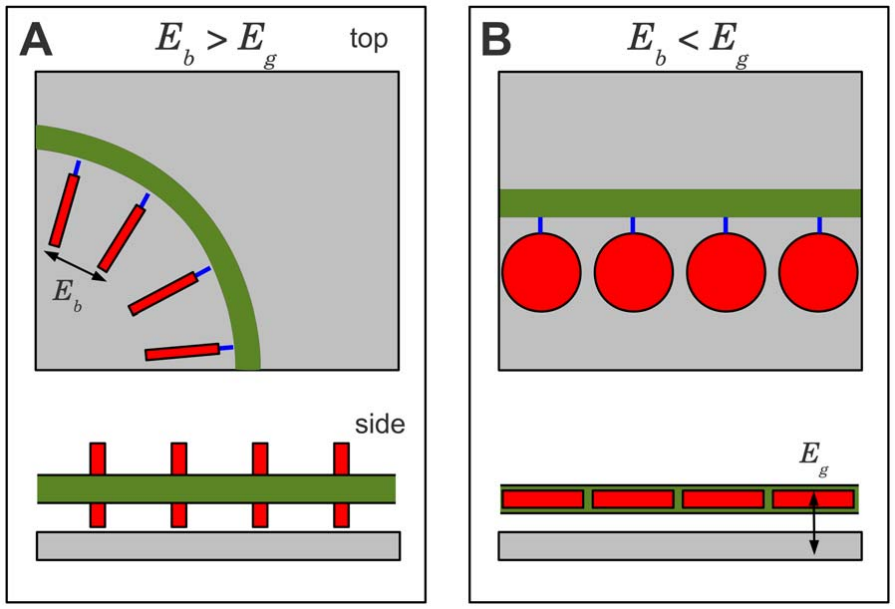
DNA assembles onto graphene (grey) into spheres (a) or networks (b). The nucleotides (red) can rotate around the link (blue) to the sugar phosphate backbone (green) to either maximize the inter-base binding energy 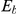 (a) or the base-graphene binding energy 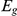 (b).

We therefore conclude that another mechanism then the ones discussed must be responsible for the observed distinct assembly behavior (figure 2). When DNA assembles onto a graphene surface, the base must rotate around the sugar-phosphate backbone to bind to the graphene, thereby breaking the base-base bond, which constitutes an energy loss 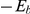. The DNA-graphene interaction provides an energy gain 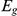. We assume that the energy required for base rotation around the link to the sugar-phosphate backbone is neglible. Here, we argue that the root cause of the DNA assembling in these distinct patterns is a crossover in the balance between these two energies, i.e. 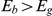 or 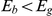. For DNA that assembles in spheres (A, C), the interbase coupling must then be more energetically favorable then the graphene interaction, 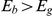 and the DNA will assemble into spherical particles to maximize 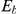 (figure 2a). For DNA assembling into networks (G, T), the graphene interaction must then be more energetically favorable, 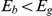. The DNA then stretches out across the graphene surface to maximize this binding energy, leading to the observed network formation (figure 2b). This hypothesis is supported by the fact that G is consistenly found to be binding most strongly to graphene, followed by A, T, and C in decreasing, but similar, binding strength (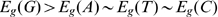) (table 1). Furthermore, the hypothesis is supported by the fact that the bond lengths for base stacking are found to be shortest for G and T and longer for A and C and the bond length is inversely related to the binding strength (table 1, last column). Hence, the inter base binding strengths is smallest for A and C, while it is larger for G and T (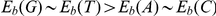). Note, however, that there is no consensus between theory and experiment; experimental studies yield relatively small binding strengths of 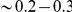 eV [22], while theoretical studies find larger binding strengths of 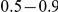 eV [17], [18]. In addition, the base-base interaction strength has not been readily measurable in experiments before. Here, the distinct assembly behavior therefore constitutes a projective measurement of the base-base interaction strength. We therefore estimate that the critical base-base interaction strength that separates network forming from spherical particle assembly is 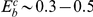 eV.

## Materials and Methods

Square highly-oriented pyrolytic graphite (HOPG) was used as a substrate (grade ZYH, Veeco, USA). The graphite was freshly cleaved with adhesive tape prior to each deposition to ensure a clean and atomically flat surface and was not further modified [23], [24]. Single-stranded DNA (ssDNA) was purchased from Integrated DNA Technologies Inc, USA. The poly-A,T, and C strands were 30 bases long, while the poly-G was 20 bases long. A 1x TAE buffer solution was prepared with 40 mM Tris-HCl, 19 mM Acetic Acid, 1 mM ethylenediaminetetraacetic acid (EDTA), and 12.5 mM Mg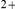. A 10 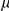
l, 0.5 mM ssDNA solution was incubated with 10 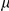
l TAE buffer and 10 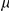
l Ni Acetate solution on the freshly-cleaved surface for 3 minutes [13]. After incubation, samples were washed with 18 M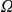 de-ionized water and dried. The graphite cleaving, ssDNA deposition, incubation, and rinsing were done at room temperature in a class 100 hood in a clean room to limit contamination of the surface. Images of the deposited DNA samples were then acquired in Close-Contact Mode with an Atomic Force Microscope (Dual-Scan AFM, Pacific Nanotechnology, USA). Control experiments were performed, in which a buffer solution without DNA was deposited and incubated on graphite, rinsed, dried, and imaged. We have used single-stranded poly DNA here to exclude the effects of hybridization.

## References

[1] Murakami H, Nomura T, Nakashima N (2003). Noncovalent porphyrin-functionalized single-walled carbon
nanotubes in solution and the formation of porphyrin-nanotube
nanocomposites.. Chemical Physics Letters.

[2] Zheng M, Jagota A, Semke ED, Diner BA, Mclean RS (2003). DNA-assisted dispersion and separation of carbon
nanotubes.. Nat Mater.

[3] Zheng M, Jagota A, Strano MS, Santos AP, Barone P (2003). Structure-Based carbon nanotube sorting by Sequence-Dependent DNA
assembly.. Science.

[4] Postma HWC (2010). Rapid sequencing of individual DNA molecules in graphene
nanogaps.. Nano Letters.

[5] Star A, Tu E, Niemann J, Gabriel JP, Joiner CS (2006). Label-free detection of DNA hybridization using carbon nanotube
network field-effect transistors.. Proceedings of the National Academy of Sciences of the United States of
America.

[6] Jeng ES, Moll AE, Roy AC, Gastala JB, Strano MS (2006). Detection of DNA hybridization using the Near-Infrared Band-Gap
fluorescence of Single-Walled carbon nanotubes.. Nano Letters.

[7] Mohanty N, Berry V (2008). Graphene-Based Single-Bacterium resolution biodevice and DNA
transistor: Interfacing graphene derivatives with nanoscale and microscale
biocomponents.. Nano Letters.

[8] Mascini M, Palchetti I, Marrazza G (2001). DNA electrochemical biosensors.. Fresenius' Journal of Analytical Chemistry.

[9] Gorodetsky AA, Barton JK (2006). Electrochemistry using Self-Assembled DNA monolayers on highly
oriented pyrolytic graphite.. Langmuir.

[10] Tang LAL, Wang J, Loh KP (2010). Graphene-Based SELDI probe with ultrahigh extraction and
sensitivity for DNA oligomer.. Journal of the American Chemical Society.

[11] Postma HWC, Sellmeijer A, Dekker C (2000). Manipulation and imaging of individual Single-Walled carbon
nanotubes with an atomic force microscope.. Advanced Materials.

[12] Penazova H, Vorlickova M (1997). Guanine tetraplex formation by short DNA fragments containing
runs of guanine and cytosine.. Biophysical Journal.

[13] Brett AMO, Chiorcea AM (2003). Atomic force microscopy of DNA immobilized onto a highly oriented
pyrolytic graphite electrode surface.. Langmuir.

[14] Brett AMO, Chiorcea A (2003). Effect of pH and applied potential on the adsorption of DNA on
highly oriented pyrolytic graphite electrodes.. atomic force microscopy surface characterisation.Electrochemistry
Communications.

[15] Brett AMO, Paquim AC (2005). DNA imaged on a HOPG electrode surface by AFM with controlled
potential.. Bioelectrochemistry.

[16] Paquim AC, Oretskaya TS, Brett AMO (2006). Atomic force microscopy characterization of synthetic pyrimidinic
oligodeoxynucleotides adsorbed onto an HOPG electrode under applied
potential.Electrochimica Acta..

[17] Gowtham S, Scheicher RH, Ahuja R, Pandey R, Karna SP (2007). Physisorption of nucleobases on graphene: Density-functional
calculations.. Physical Review B.

[18] Antony J, Grimme S (2008). Structures and interaction energies of stacked
graphene-nucleobase complexes.. Physical Chemistry Chemical Physics.

[19] Varghese N, Mogera U, Govindaraj A, Das A, Maiti PK (2009). Binding of DNA nucleobases and nucleosides with
graphene.. Chemphyschem: A European Journal of Chemical Physics and Physical
Chemistry.

[20] Chiorcea-Paquim AM, Oretskaya TS, Brett AMO (2006). Adsorption of synthetic homo-and hetero-oligodeoxynucleotides
onto highly oriented pyrolytic graphite: Atomic force microscopy
characterization.. Biophysical chemistry.

[21] Saenger W (1984). Principles of Nucleic Acid Structure..

[22] Manohar S, Mantz AR, Bancroft KE, Hui C, Jagota A (2008). Peeling Single-Stranded DNA from graphite surface to determine
oligonucleotide binding energy by force spectroscopy.. Nano Letters.

[23] Adamcik J, Klinov DV, Witz G, Sekatskii SK, Dietler G (2006). Observation of single-stranded DNA on mica and highly oriented
pyrolytic graphite by atomic force microscopy.. FEBS Letters.

[24] Tang Z, Wu H, Cort JR, Buchko GW, Zhang Y (2010). Constraint of DNA on functionalized graphene improves its
biostability and specificity.. Small.

[25] Matta CF, Castillo N, Boyd RJ (2006). Extended weak bonding interactions in DNA: pi-Stacking
(Base-Base), Base-Backbone, and Backbone-Backbone
interactions.. The Journal of Physical Chemistry B.

